# Estimation of SARS-CoV-2 IgG Antibodies in Healthcare Worker-Administered Covishield and Covaxin Vaccines at a Tertiary Care Hospital in Jharkhand, India

**DOI:** 10.7759/cureus.47566

**Published:** 2023-10-24

**Authors:** Bishnupati Singh, Kumari Seema, Amit V Mahuli, Abhay Kumar, Manju Boipai, Ashok K Sharma, Manoj Kumar, Surender Kumar, Subhash Chandra, Ajoy K Shahi

**Affiliations:** 1 Department of Prosthodontics, Dental Institute, Rajendra Institute of Medical Sciences, Ranchi, IND; 2 Department of Microbiology, Rajendra Institute of Medical Sciences, Ranchi, IND; 3 Department of Public Health Dentistry, Dental Institute, Rajendra Institute of Medical Sciences, Ranchi, IND; 4 Department of Orthodontics and Dentofacial Orthopaedics, Dental Institute, Rajendra Institute of Medical Sciences, Ranchi, IND; 5 Department of Oral and Maxillofacial Surgery, Dental Institute, Rajendra Institute of Medical Sciences, Ranchi, IND

**Keywords:** sara-cov-2, igg antibodies, covishield, covaxin, covid-19 india

## Abstract

Introduction

To mitigate the impact of the COVID-19 pandemic caused by the SARS-CoV-2 virus, global distribution of vaccines such as Covishield and Covaxin has been undertaken. This research aimed to assess the responses and potential differences between these vaccines by examining the presence and levels of SARS-CoV-2 IgG antibodies in healthcare professionals who received them.

Methodology

A comprehensive cross-sectional study was conducted at a tertiary care facility in Ranchi involving 227 healthcare professionals who had completed both doses of either Covishield or Covaxin. Blood samples were collected and subjected to chemiluminescence immunoassay analysis to measure IgG antibodies. Demographic data, immunization records, and previous COVID-19 infections were recorded. Statistical analyses, including analysis of variance (ANOVA), linear regression, and independent sample t-tests were performed.

Results

Antibody titers exhibited variability, potentially influenced by factors. There was no difference in antibody titers between recipients of Covishield and Covaxin vaccines. Linear regression analysis revealed a correlation between antibody levels and the number of days after vaccination. Factors such as age, gender, blood group, and prior COVID-19 infections did not significantly impact antibody titers.

Conclusions

This study contributes to responses elicited by Covishield and Covaxin vaccines among healthcare workers. The results highlight that Covishield showed a higher mean titer value than Covaxin, which is not statistically significant. The overall model showed statistically significant results indicating age, type of vaccine, number of days after vaccination, blood group, and previous history of COVID-19 infection collectively influenced the CoV-2 IgG titer values. The findings indicate that age, number of days after vaccination, and prior history of COVID-19 infection have substantial relationships with the CoV-2 IgG titer, but sex, vaccine type, and blood group show lesser, nonsignificant associations.

## Introduction

The first COVID-19 case was reported in December 2019 in Wuhan, Hubei, China, as pneumonia of unknown origin. SARS-CoV-2 was eventually established as the cause of the pneumonia. The sickness was later labeled a pandemic by the World Health Organization in March 2020, making it one of the most feared diseases in human history [1]. As of August 9, 2023, there were 44,996,059 confirmed cases of COVID-19 in India, with 531,918 deaths, and a total of 2,206,755,904 vaccine doses had been delivered [2]. COVID-19 vaccinations come in a variety of forms, including viral vector vaccines, protein subunit vaccines, genetic vaccines, and monoclonal antibodies for passive immunization [3].

In India, the vaccination drive started on January 16, 2021, with Covishield (a version of the Oxford University-AstraZeneca vaccine) and later Covaxin (Bharat Biotech, ICMR, and NIV) was added and are widely in use while others are being considered for emergency use to cater to 1.4 billion plus population [4]. The second wave of COVID-19 in India severely affected the population and healthcare workers with over 4 lakh cases per day at its peak [5]. Hence, it becomes vital to understand the vaccine’s effectiveness in controlling the SARS-CoV-2 infection.

The emergence of SARS-CoV-2 variants, such as the Delta variant, has raised concerns about vaccine efficacy. Recent research indicates that Covishield retains significant protection against severe outcomes even in the face of Delta variant circulation [6]. Similarly, Covaxin has demonstrated efficacy against multiple variants [7]. Nevertheless, it is essential to determine the level of neutralizing antibodies generated by these vaccines against the Delta variant and other variants of concern.

Antibodies are indicators of a person's immune system's ability to battle viral infections and play an important part in the immunological response to viral infections. SARS-CoV-2-specific IgG antibodies are thought to be an important predictor of viral immunity. As a result, assessing the existence and quantity of SARS-CoV-2 IgG antibodies in healthcare professionals who have received either the Covishield or Covaxin vaccinations is critical for understanding the vaccine's efficacy and guiding immunization strategy [8].

Thus, the study aimed to estimate the SARS-CoV-2 IgG antibodies among the healthcare workers administered with Covishield and Covaxin at a tertiary care hospital in Ranchi. This will guide us to understand the SARS-CoV-2 IgG antibodies in healthcare workers with a natural history of COVID-19 infection and vaccination variations.

## Materials and methods

Study design

The study was a descriptive cross-sectional study to estimate the SARS-CoV-2-specific IgG antibody in healthcare workers of a tertiary care hospital in Ranchi.

Study area and population

The study was conducted among the healthcare workers of Rajendra Institute of Medical Sciences (RIMS), Ranchi. The list of healthcare workers who received both doses of vaccine and completed 21 days was collected. The sample of 227 individuals vaccinated with Covishield and Covaxin, who met the eligibility criteria and provided consent to participate in the study, was considered.

Study period

The study was conducted for three months (December 9, 2021, to February 8, 2022) following ethical approval.

Sample size

Recent studies have reported a seroconversion of 85% in the Indian population [9]. The minimum necessary sample size was determined to be 196 patients to evaluate a similar proportion of 95% with a precision estimate of 0.05 at a confidence level of 95%.

Eligibility criteria

All the healthcare workers who have taken both doses of vaccination were included in the study. All the subjects willing to participate in the study and those who gave voluntary consent were included. The subjects with immunocompromised status and other known medical conditions influencing the IgG levels were excluded from the study. Individuals with current confirmed SARS-CoV-2 infection on the day of sample collection and not willing to participate were excluded from the study.

Data collection

As marker/demographics name, age, sex, and address of all the participants were noted. The blood sample of the consented individuals was collected, and the presence of IgG antibodies was noted.

Quantitative variables

The proforma will be used to collect the demographic details, the status of vaccination, type of blood group, co-morbidities, history of previous COVID-19 infection, type of vaccine administered, and quantitative value of results of IgG antibodies recorded. Three milliliters of blood samples were collected in ethylenediaminetetraacetic acid (EDTA) vials from COVID-19-vaccinated healthcare workers who agreed to give samples and analyzed them in the Department of Microbiology, RIMS, Ranchi.

First, 50 µL of whole blood was drawn, and blood grouping was performed using the slide agglutination method and according to the manufacturer's instructions. Following that, blood samples were centrifuged at 2500 rpm for 10 minutes, and plasma was separated for quantitative detection of IgG antibodies to the nucleocapsid protein of SARS-CoV-2 virus using chemiluminescence immunoassay (CLIA) with SARS-CoV-2 IgG detection kit (Architect, Abbott Laboratories, Abbott Park, IL). In an automated CLIA system, a mixture of SARS-CoV-2 antigen-coated paramagnetic microparticles, assay diluent, and 150 µl of plasma sample was incubated. The remaining plasma samples were stored in a deep freezer for later use.

The SARS-CoV-2 IgG antibodies found in the sample bind to the antigen-coated microparticles. After washing the mixture, the anti-human IgG acridinium-labeled conjugate was added to create a reaction mixture, which was subsequently incubated. After a wash cycle, pre-trigger and trigger solutions were applied. The chemiluminescent reaction that resulted was assessed as relative light unit (RLU), and there was a direct association between the level of IgG antibodies to SARS-CoV-2 in the sample and the RLU detected by the system optics. As a result, IgG antibody titers were calculated against the corresponding vaccination (Covaxin or Covishield), and seropositivity was defined as 50 arbitrary units (AU)/mL or greater.

Statistical analysis

The obtained data were analyzed for descriptive statistics, and frequency distribution was conducted. The normality of the data was checked with the Shapiro-Wilk test. Frequencies were compared using the Chi-square test. The Covishield and Covaxin antibody titters were compared using an independent sample t-test. A linear regression analysis was conducted to examine the relationship between several predictors and the CoV-2 IgG titer, followed by ANOVA. The IBM SPSS Statistics for Windows, Version 26.0 (IBM Corp., Armonk, NY) software was used to perform the statistical analysis. *P*-value < 0.05 was considered significant.

## Results

A total of 227 participants were included in the study, with a mean age of 38.7 ± 11.3 years (standard deviation, SD). The study participants constituted 67% (152) male and 33% (75) female. The age was grouped into five categories (Table 1).

**Table 1:** Age of the study participants grouped in different categories, study participants vaccinated with Covaxin and Covishield, number of days after vaccination of the study participants grouped in different categories, different types of blood groups distribution in study participants, and the previous history of COVID-19 infection.

Groups	Frequency, *n*	Percentage (%)
Type of vaccination		
Covaxin	92	40.5
Covishield	135	59.5
Total	227	100
Age (years)		
Less than or equal to 30	65	28.6
31-40	75	33
41-50	52	22.9
51-60	24	10.6
Greater than or equal to 61	11	4.8
Total	227	100
Number of days after vaccination		
Less than or equal to 100 days	6	2.6
101-200 days	18	7.9
201-300 days	98	43.2
301-400 days	98	43.2
Greater than or equal to 401 days	7	3.1
Total	227	100
Blood group		
O+	75	33
A+	49	21.6
AB+	18	7.9
B+	80	35.2
AB-	3	1.3
B-	2	0.9
Total	227	100
Previous history of COVID-19 infection		
Before the first dose	40	17.6
After the first dose before the second dose	8	3.5
After the second dose	41	18.1
Not infected	132	58.1
Multiple infections of COVID-19	6	2.6
Total	227	100

The number of days after vaccination showed a mean (SD) of 291.64 ± 91.13. The mean value of antibody titer was 4631.5 AU/mL and an SD of 7813.19 AU/mL, thus showing a very high variability of data. The difference between the titer values of Covaxin and Covishield was calculated using an independent sample t-test (Table 2). The results showed no statistical significance; however, Covishield showed a higher mean titer value than Covaxin.

**Table 2:** Difference between the titer values of Covaxin and Covishield. *F*, Fisher value; Sig., significance level; CI, confidence interval

Type of vaccine	Number	Mean	Standard deviation	F	Sig.	95% CI of the difference	
Covaxin	92	4,359	7,000	-0.433	0.666	Lower	Upper
						-2,543	1,627
Covishield	135	4,817	8,342			-2,475	1,559

Covaxin showed a seropositivity of 89.13%, and Covishield showed a seropositivity of 95.5% (Table 3).

**Table 3:** Seropositivity of Covaxin and Covishield.

Type of vaccine	Seropositivity	Counts	Percentage of seropositivity (%)
Covaxin	Negative	10	10.87
	Positive	82	89.13
Covishield	Negative	6	4.5
	Positive	129	95.5

A linear regression analysis was conducted to examine the relationship between several predictors and the CoV-2 IgG titer. The model aimed to investigate the impact of age, sex, type of vaccine, number of days after vaccination, blood group, and previous history of COVID-19 infection on the CoV-2 IgG titer. The results of the analysis are presented in Table 4.

**Table 4:** Linear regression analysis of predictors: (Constant), previous history of COVID-19 infection, blood group, type of vaccine, sex, number of days after vaccination, and age. *B*, coefficients; Std. error, standard error; *t*, test value; Sig., significance

Model	Unstandardized coefficients		Standardized coefficients	t	Sig.	95% CI for *B*	
	B	Std. error	Beta			Lower bound	Upper bound
(Constant)	-10089.21	3785.45		-2.665	0.008	-17549.59	-2628.81
Age	136.17	481.73	0.02	0.283	0.778	-813.23	1085.58
Sex	1782.76	1122.69	0.108	1.588	0.114	-429.855	3995.38
Type of vaccine	664.79	1052.9	0.042	0.631	0.528	-1410.26	2739.85
No. of days after vaccination	2503.88	688.78	0.251	3.635	0.001	1146.43	3861.34
Blood group	396.16	377.69	0.068	1.049	0.295	-348.20	1140.54
Previous history of COVID-19 infection	479.78	443.55	0.072	1.082	0.281	-394.37	1353.94

The analysis revealed that age (*β* = 0.020, *t* = 0.283, and *P* = 0.778), sex (*β* = 0.108, *t* = 1.588, and *P* = 0.114), type of vaccine (*β* = 0.042, *t* = 0.631, and *P* = 0.528), number of days after vaccination (*β* = 0.251, *t* = 3.635, and *P* < 0.001), blood group (*β* = 0.068, *t* = 1.049, and *P* = 0.295), and previous history of COVID-19 infection (*β* = 0.072, *t* = 1.082, and *P* = 0.281) were included as predictors in the regression model.

The constant term (*β* = -10,089.205, *t* = -2.665, and *P* = 0.008) represents the intercept value of the CoV-2 IgG titer when all predictor variables are zero. It indicates the baseline value of the CoV-2 IgG titer in the absence of the included predictors.

The overall model fit was statistically significant (*F* = 3.429, *P* = 0.003), indicating that the predictors collectively contributed to the prediction of the CoV-2 IgG titer. The model explained a significant proportion of the variance in the CoV-2 IgG titer, as indicated by the coefficient of determination (*R*^2^ = 0.292). The adjusted coefficient of determination (adjusted *R*^2^ = 0.086) accounts for the degrees of freedom and adjusts the *R*^2^ value for the number of predictors in the model, providing a more accurate estimate of the model's explanatory power (Table 5).

**Table 5:** The overall model summary of the predictors in linear regression analysis: (Constant), previous history of COVID-19 infection, blood group, type of vaccine, sex, no. of days after vaccination, and age. *R*, linear regression correlation coefficient; *R*^2^, coefficient of determination; Std. error, standard error; *F*, *F*-test value; df, degree of freedom; Sig., significance

Model	R	*R*^2^	Adjusted *R*^2^	Std. error of the estimate	Change statistics				
					*R*^2 ^change	*F* change	df1	df2	Sig. *F* change
	0.292	0.086	0.061	7572.8773	0.086	3.429	6	220	0.003

These findings indicate that age, number of days after vaccination, and prior history of COVID-19 infection have substantial relationships with the CoV-2 IgG titer, but sex, vaccine type, and blood group show lesser, nonsignificant associations. To completely comprehend the individual and combined effects of these predictors on the CoV-2 IgG titer, more research and interpretation are required. The ANOVA test was applied to examine differences among the IgG antibody titers based on age, previous history of COVID-19, number of days after vaccination, and blood group. The results showed no statistical significance (Table 6).

**Table 6:** Previous history of COVID-19 infection and CoV-2 IgG titer, age and CoV-2 IgG titer, number of days after vaccination and CoV-2 IgG titer, and blood group and CoV-2 IgG titer: ANOVA test. *F*, *F*-value; Sig., significance; ANOVA, analysis of variance; Std. deviation, standard deviation

Previous history of COVID-19 infection							
	Number	CoV-2 IgG titer (Mean)	CoV-2 IgG titer (Std. deviation)	95% CI for mean			
				Lower bound	Upper bound	F	Sig.
Before the first dose	40	2378.5	3022.3445	1411.905	3345.09	1.032	0.391
After the first dose before the second dose	8	4952.14	7243.3073	-1103.42	11007.69		
After the second dose	41	5237.34	5950.245	3359.208	7115.466		
Not infected	132	5047.58	9192.56	3464.775	6630.39		
Multiple infections of COVID-19	6	5930.57	8183.6345	-2657.63	14518.76		
Total	227	4631.51	7813.1912	3609.637	5653.377		
Age (years)							
Less than or equal to 30	65	4926.85	7196.0186	3143.762	6709.934	0.972	0.424
31-40	75	3296.36	5595.0669	2009.051	4583.667		
41-50	52	5142.43	8651.8091	2733.746	7551.104		
51-60	24	6145.91	11210.312	1412.209	10879.61		
Greater than or equal to 61	11	6270.2	11046.455	-1150.91	13691.31		
Total	227	4631.51	7813.1912	3609.637	5653.377		
Number of days after vaccination							
Less than or equal to 100 days	6	4797.63	4035.6569	562.471	9032.795	1.032	0.391
101-200 days	18	1773.84	3094.1938	235.138	3312.551		
201-300 days	98	2119.08	4493.979	1218.09	3020.061		
301-400 days	98	7565.26	9425.6614	5675.534	9454.985		
Greater than equal to 401 days	7	5938.9	15023.382	-7955.41	19833.21		
Total	227	4631.51	7813.1912	3609.637	5653.377		
Blood group							
O+	75	3890.23	5775.6488	2561.369	5219.081	0.271	0.929
A+	49	4667.39	8754.7526	2152.731	7182.048		
AB+	18	5061.14	7293.5966	1434.124	8688.165		
B+	80	5116.48	9170.2403	3075.747	7157.221		
AB-	3	4947.37	2929.83	-2330.73	12225.47		
B-	2	7810.85	285.7419	5243.561	10378.14		
Total	227	4631.51	7813.1912	3609.637	5653.377		

## Discussion

The use of Covishield and Covaxin vaccines at a tertiary care hospital among healthcare professionals in Ranchi shows the immune response elicited by these vaccines and provides insights into the dynamics of antibody production within this specific cohort. The demographic characteristics of the study participants revealed a diverse representation in terms of age, gender, blood groups, and previous history of COVID-19 infection. The gender distribution mirrored the healthcare workforce's composition, while the age distribution spanned a broad range, reflecting the varied age groups of healthcare professionals. This diversity adds depth to the study's generalizability and allows for insights into potential age-related variations in immune response.

The analysis of antibody titers demonstrated considerable variability among the study participants. This observation aligns with previous studies, highlighting the heterogeneity of immune responses following vaccination [7,9-11]. Factors such as genetics, prior exposure to SARS-CoV-2, and individual variations in immune systems might contribute to this wide range of antibody titers [9-11].

The Covishield vaccine is a viral vector-based vaccination, also known as the ChAdOx1 nCoV-19 Corona Virus Vaccine. It employs a modified adenovirus vector to transmit the genetic material responsible for the SARS-CoV-2 virus's spike protein. The Covishield vaccine, when administered, induces the immune system to recognize and develop antibodies against the spike protein, protecting against SARS-CoV-2 infection. BBV-152 Covaxin, on the other hand, is an inactivated whole-virus vaccine. It contains inactivated SARS-CoV-2 virus particles, which cannot replicate or cause disease but can still stimulate an immune response. The study aimed to compare the seropositivity rates of anti-spike IgG antibodies among healthcare workers administered with either Covishield or Covaxin [7,9,11,12].

The independent sample t-test comparing the antibody titers between Covishield and Covaxin recipients showed no statistically significant difference. This suggests that, within the parameters of this study, both vaccines elicited similar antibody responses among the healthcare workers with a slightly higher mean value for Covishield. However, it's essential to note that this finding should be interpreted with caution, as vaccine responses encompass a broader spectrum of immunological markers beyond IgG antibodies. Covishield showed higher seropositivity, which was similar to the study results where Covishield showed better results than Covaxin [12,13]. The study results of another study showed contrasting results where Covaxin showed better results than Covishield [9].

The linear regression analysis aimed to identify predictors influencing the CoV-2 IgG antibody titer. Among the examined predictors, the number of days after vaccination exhibited a significant positive association with the antibody titer. This finding is consistent with the expected kinetics of immune response, where antibody levels tend to increase over time following vaccination. This temporal relationship emphasizes the importance of allowing adequate time for the immune system to mount a robust response.

Some study findings show that after receiving the COVID-19 vaccine, IgG antibody responses are still strong and persistent, with greater titers in previously exposed individuals and lasting at least 12 months in most patients, and vaccine-induced immunity is more effective than infection-induced immunity [14].

Interestingly, age demonstrated a nonsignificant association with antibody titer in this cohort. Although age-related differences in immune response have been reported, the lack of significance in this study could be attributed to the relatively small sample sizes within specific age groups. Further research with larger samples may offer insights into potential age-dependent antibody responses [15].

ANOVA tests exploring the impact of blood group and previous history of COVID-19 infection on antibody titers revealed no statistically significant associations. These findings suggest that neither blood group nor prior infection significantly influenced antibody levels within this cohort of healthcare workers. The results of some studies show a higher risk for blood group A, our study showed a relatively lesser mean value for IgG levels showing the risk [16]. However, it's important to recognize that the relationship between blood group and COVID-19 immunity is complex and multifactorial, involving both genetic and immunological factors.

This study is not without limitations. The cross-sectional design restricts our ability to establish causality, and the relatively small sample size could limit the generalizability of the findings to other populations. Additionally, the study's focus on IgG antibodies provides only a partial view of the immune response. Future investigations could include a broader range of immune markers, such as neutralizing antibodies and T-cell responses. However, there is a strong correlation between the total and IgG anti-spike antibodies with neutralizing antibodies [14].

Despite these limitations, this study contributes valuable insights into the antibody response following Covishield and Covaxin vaccination among healthcare workers. The absence of a significant difference between the two vaccines underscores their potential to induce comparable immune responses. Further longitudinal studies with larger cohorts are warranted to assess the durability of antibody responses and their correlation with vaccine efficacy and protection against SARS-CoV-2 infection.

## Conclusions

In conclusion, this study enhances our understanding of the immune response to COVID-19 vaccines in a specific healthcare worker population. The results highlight that Covishield showed a higher mean titer value than Covaxin, which is not statistically significant. The overall model showed statistically significant results indicating age, type of vaccine, number of days after vaccination, blood group, and previous history of COVID-19 infection collectively influenced the CoV-2 IgG titer values. The findings indicate that age, number of days after vaccination, and prior history of COVID-19 infection have substantial relationships with the CoV-2 IgG titer, but sex, vaccine type, and blood group show lesser, nonsignificant associations. Further research into the factors influencing vaccine-induced immunity is recommended. As the pandemic landscape evolves, such investigations are vital for refining vaccination strategies and optimizing public health interventions.
